# Hydrothermal‐Assisted In Situ Intercalation Synthesis of Ti_3_C_2_T_x_ MXene in 6 Hours

**DOI:** 10.1002/smtd.70871

**Published:** 2026-07-28

**Authors:** Muhammad Imran Rameel, Gediminas Monastyreckis, Filipa M. Oliveira, Bing Wu, Jiří Šturala, Vlastimil Mazánek, Zdeněk Sofer, Daiva Zeleniakiene

**Affiliations:** ^1^ Department of Mechanical Engineering Kaunas University of Technology Kaunas Lithuania; ^2^ Department of Inorganic Chemistry Faculty of Chemical Technology University of Chemistry and Technology Prague Prague Czech Republic

**Keywords:** 2D materials, hydrothermal‐assisted etching, in situ intercalation, Ti_3_C_2_T_x_ MXene

## Abstract

Despite the rapid expansion of MXene research, scalable and time‐efficient synthesis routes that simultaneously deliver high‐quality, high‐yield products remain limited. Here, we present a facile, high‐yield method for the synthesis, in situ intercalation, and delamination of Ti_3_C_2_T_x_ MXene. In situ intercalation during hydrothermal‐assisted etching facilitates aluminum removal and promotes the delamination of MXene sheets. This approach reduces synthesis time to 6 h at 90°C, compared to up to 48 h in conventional protocols. The methodology employs a mild etchant system of hydrochloric acid and lithium fluoride, with lithium chloride as an in situ intercalant within a closed hydrothermal autoclave. Hydrothermal conditions enhance etchant reactivity and kinetics, while Li^+^ ions weaken van der Waals forces, enabling exfoliation into two‐dimensional MXenes. Comprehensive characterization confirms the method's high efficacy, yielding 74.2% ± 3.7% delaminated nanosheets with a monolayer thickness of 1–1.5 nm. X‐ray diffraction confirms successful Al etching and effective delamination. X‐ray photoelectron and energy‐dispersive spectroscopies show surface terminations consisting of –Cl, –F, and –O. The resulting MXene exhibits an electrical conductivity of 7 500 S cm^−^^1^. This methodology offers a scalable, single‐step route to MXene synthesis, marking a significant advance and opening avenues for commercial applications of these materials.

## Graphical abstract

This work presents a rapid, scalable strategy for synthesizing Ti_3_C_2_T_x_ MXene through hydrothermal‐assisted etching with in situ intercalation, achieving complete synthesis and delamination within 6 h at 90°C. The process accelerates aluminum removal, promotes efficient delamination, and enables direct exfoliation under mild conditions, producing high‐quality MXene nanosheets with consistent structural and chemical properties, offering a route toward scalable production.

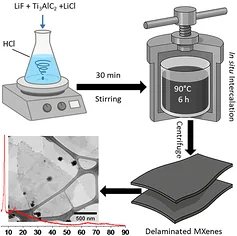

## 1 Introduction

MXenes are two‐dimensional (2D) transition‐metal carbides, nitrides, or carbonitrides that have attracted significant attention since their discovery in 2011 [1]. Their remarkable properties, including high electrical conductivity, hydrophilicity, tunable surface chemistry, and excellent mechanical stability [2, 3] position them as promising materials for a wide array of applications such as energy storage, sensors, solar cells, catalysis, and antimicrobial coatings [4, 5]. Ti_3_C_2_T_x_ (Ti‐MXene) is the most studied MXene [1] historically synthesized through a two‐step process involving the etching of Ti_3_AlC_2_ MAX phase using etchants like hydrofluoric acid (HF), followed by delamination to obtain separated 2D sheets [6, 7]. Despite its prevalence, this traditional approach faces significant challenges, including low yields, often reported below 20% [8, 9] due to strong interlayer interactions, such as potential Ti−Ti bonds (1.07 eV) and residual Ti−Al bonds (2.26 eV), which hinder effective intercalation and delamination [10]. Moreover, concentrated HF (∼50%) is highly toxic, making it challenging to handle and dispose of the waste. The hazards associated with handling HF make it challenging to scale up the MXene synthesis process to a commercial level [11]. To replace hazardous HF in MXene synthesis [12], introduced ammonium bifluoride (NH_4_HF_2_) as a safer etchant. Building on this, Ghidiu et al. developed a milder HCl/LiF approach to suppress direct HF reactions [13]; however, the process requires longer etching time (up to 48 h). Moreover, existing methods often yield multilayer MXenes, necessitating an additional, usually time‐consuming, intercalation and delamination step to separate them into mono‐ or few‐layer sheets [14, 15]. Delamination is typically achieved through chemical intercalation using salts (such as LiCl, NaCl, or KCl) [16] or organic solvents (such as dimethyl sulfoxide [17], urea, hydrazine, and tetramethylammonium hydroxide) [18], often followed by agitation methods such as shaking or sonication. Among these intercalants, LiCl is widely used, particularly when etching conditions are optimized to increase the interlayer spacing [19]. Hydrothermal assistance is a great way to isolate harmful etchants and gases in a closed container, and it also reduces the hazards associated with HF [20, 21]. This process also increases the ionization constant of water by decreasing its viscosity and surface tension, effectively resolving solvation issues for etching agents like LiF [22]. Additionally, the vibration of Li^+^ ions, enhanced by hydrothermal heating and increased ionic mobility, facilitates their intercalation into the Ti_3_AlC_2_ sheets, leading to the delamination of the MXene sheets after Al removal [22, 23]. Moreover, hydrothermal‐assisted intercalation (HAI) strategies have demonstrated a significant increase in the yield of 2D Ti_3_C_2_T_x_ sheets, reaching 74% [24].

Herein, we report a method for synthesizing Ti_3_C_2_T_x_ MXene from the Ti_3_AlC_2_ MAX phase that combines hydrothermal assistance, mild etching, and in situ intercalation. Crucially, hydrothermal assistance enables the direct production of single‐layer Ti_3_C_2_T_x_ sheets in reduced time, followed by centrifugation and washing steps, skipping the conventional, separate delamination process typically required to achieve high yields of exfoliated sheets. This approach achieves a 74.2% ± 3.7% yield and an electrical conductivity of 7 500 S cm^−^^1^ in a remarkably reduced synthesis time (6 h instead of the typical 48 h). The resulting material is obtained and stored directly as a suspension. This work presents a significant advancement in the synthesis of Ti_3_C_2_T_x_, offering a facile, rapid, and high‐yield one‐step route to single‐layered MXene suspensions by integrating etching and delamination steps, potentially paving the way for more efficient and scalable production as compared to other existing synthesis methods of MXene such as HF etching [25, 26], in situ HF etching [27], electrochemical etching [28], and molten salts etching [29].

## 2 Results and Discussion

In this work, the transformation of the Ti_3_AlC_2_ MAX phase into the Ti_3_C_2_T*_x_* MXene was achieved via facile, mild hydrothermal synthesis with in situ intercalation. Under these conditions, delaminated Ti_3_C_2_T_x_ was obtained after only 6 h at 90°C using LiF/HCl with LiCl as the in situ intercalant, thereby avoiding the prolonged post‐etching delamination step typically required in the conventional MILD method. In comparison, the typical MILD route commonly requires etching up to 48 h, followed by an additional delamination step that may last up to 18 h [30]. The yield of the synthesized MXene was calculated by measuring mg of MXene per mL of MXene suspension. MXene suspension (3.6 L) was collected from 2 g of MAX phase precursor. The MXene concentration was determined by drying 20 mL of MXene suspension. A concentration of 401 µg/mL was obtained, corresponding to a yield of 72.1% [31]. To assess the reproducibility of the proposed synthesis route, the yield was determined across four independent batches, resulting in an average single‐ or few‐layer Ti_3_C_2_T*_x_* MXene yield of 74.2% ± 3.7%. The synthesized Ti_3_C_2_T*_x_* MXene has been investigated in terms of structural, morphological, optical, and chemical properties.

**FIGURE 1:**
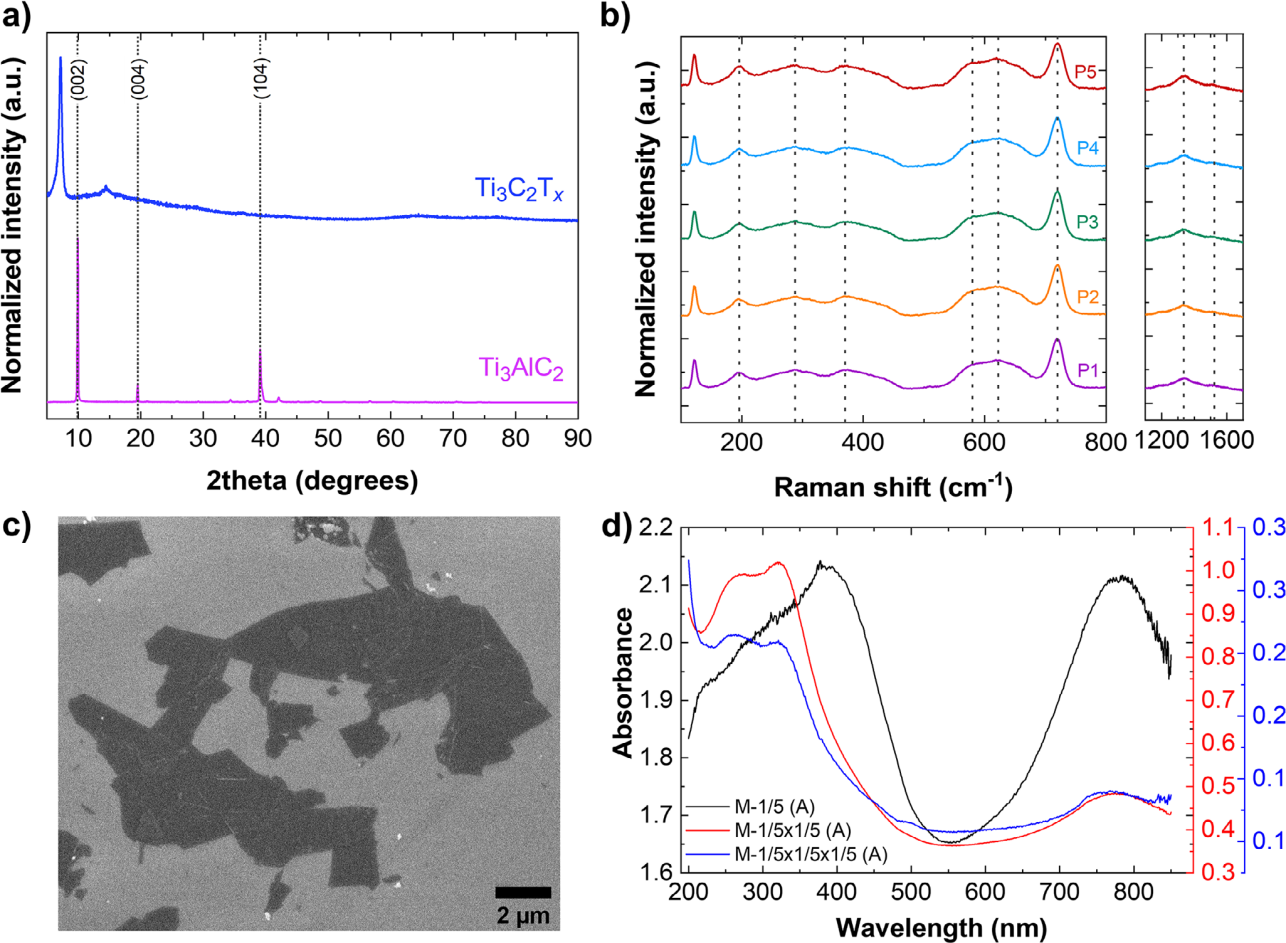
Characterization of Ti_3_C_2_T*_x_* 2D sheets synthesized via the hydrothermally assisted in situ intercalation strategy, 90°C for 6 h: (a) XRD patterns of the Ti_3_C_2_T*_x_* MXene and parent Ti_3_AlC_2_ MAX phase, (b) Raman spectra of Ti_3_C_2_T*_x_* MXene collected at five different positions, (c) SEM micrograph of prepared Ti_3_C_2_T*_x_* flakes, and (d) UV‐Visible absorption curves at high and low concentrations.

The XRD patterns of the Ti_3_AlC_2_ MAX phase and the corresponding delaminated MXene are shown in Figure 1a. The MAX phase exhibits the characteristic (104) reflection at 39.1°, which disappears after etching, confirming the successful removal of the Al layers. In the MXene pattern, the characteristic (002) and (004) reflections are observed at 7.2° and 14.5°, respectively, confirming the layered structure of Ti_3_C_2_T*_x_* MXene [32]. The (002) peak is significantly sharp and narrow, further suggesting a high degree of structural order and effective delamination. The interlayer *d*‐spacing, calculated from Bragg's law, is 12.23 Å. The observed shift towards a higher interlayer spacing than in the MAX phase (9.29 Å) [33] and the disappearance of the MAX phase characteristic reflection (104) at 39.1° indicates complete etching of Al and transformation into Ti_3_C_2_T*_x_*. The *d*‐spacing is even higher than the published value for the HF protocol (9.8 Å) [34] and is similar to that of other Li^+^‐intercalated MXenes (about 11.5 Å) [35], supporting the occurrence of in situ Li^+^ intercalation during the hydrothermal process.

The observed increase in interlayer spacing and the absence of residual MAX‐phase reflections indicate that the hydrothermal LiF/HCl route promotes not only selective Al removal but also simultaneous intercalation‐assisted delamination. This interpretation is consistent with previous reports showing that closed hydrothermal processing improves MXene structural development and exfoliation behavior compared with open‐system treatment [36, 37]. Temperature‐dependent studies on LiF/HCl etching have shown that increasing temperature enhances Al removal, although longer treatment times are still required in an open vessel [38]. In addition, when LiCl is present during etching, Li^+^ ions are introduced into the interlayer space of Ti_3_C_2_T*_x_*, thereby increasing structural order and enabling direct delamination without a separate post‐etching intercalation step [39]. This is consistent with present results and supports the interpretation that Li^+^‐assisted intercalation contributes to the shortened overall processing route under hydrothermal conditions in an enclosed environment. The short reaction time of 6 h used here is also consistent with the literature, which shows that Ti_3_AlC_2_ etching exhibits a strong temperature dependence and can be described by nucleation‐and‐growth‐based kinetic models with apparent activation energies of about 54–56 kJ mol^­1^, indicating that even a modest increase in temperature can substantially shorten the MXene synthesis time by accelerating the etching process [40].

Complementary to the XRD analysis, the Raman spectra shown in Figure 1b are consistent with previous studies on the vibration modes of Ti_3_C_2_T*_x_* MXenes [41, 42]. Typical vibrations related to titanium *A*_1_ *_g_* at 196 cm^−1^ and broad peaks at 288 cm^−1^ and 370 cm^−1^ assigned to in‐plane *E_g_* modes of surface groups attached to Ti atoms are identified. In the so‐called carbon region (550–750 cm^−1^), the peaks observed at 579 cm^−1^, 621 cm^−1,^ and 718‐720 cm^−1^ are assigned to Ti (*E_g_*) and C (*A*_1_ *_g_*) vibrations, respectively. A distinct peak around 121 cm^−1^ is also observed. This mode has been reported to exhibit resonant behavior under 785 nm excitation [41], which is consistent with the laser used in this work. Additionally, the carbon defect region (1200–1800 cm^−1^) was included, where broad peaks at ∼1328 cm^−1^ and ∼1520 cm^−1^ can be identified and are associated with disordered and graphitic carbon species.

The morphological features of the obtained single‐layer Ti_3_C_2_T*_x_* MXene were observed by SEM, as shown in Figure 1c. The image reveals flat, sheet‐like flakes that are clearly separated, indicating successful delamination from the multilayered Ti_3_AlC_2_ MAX phase precursor. Unlike multilayer MXenes, which typically exhibit an accordion‐like morphology after etching, the observed flakes appear isolated, as thin sheets with sharp hexagonal edges and sub‐micrometer lateral size. This morphology is characteristic of delaminated Ti_3_C_2_T*_x_* nanosheets [34]. Figure S2 shows a SEM image of the leftover paste, which is a few‐ or multi‐layered MXene, confirming the complete etching of the MAX phase.

**FIGURE 2:**
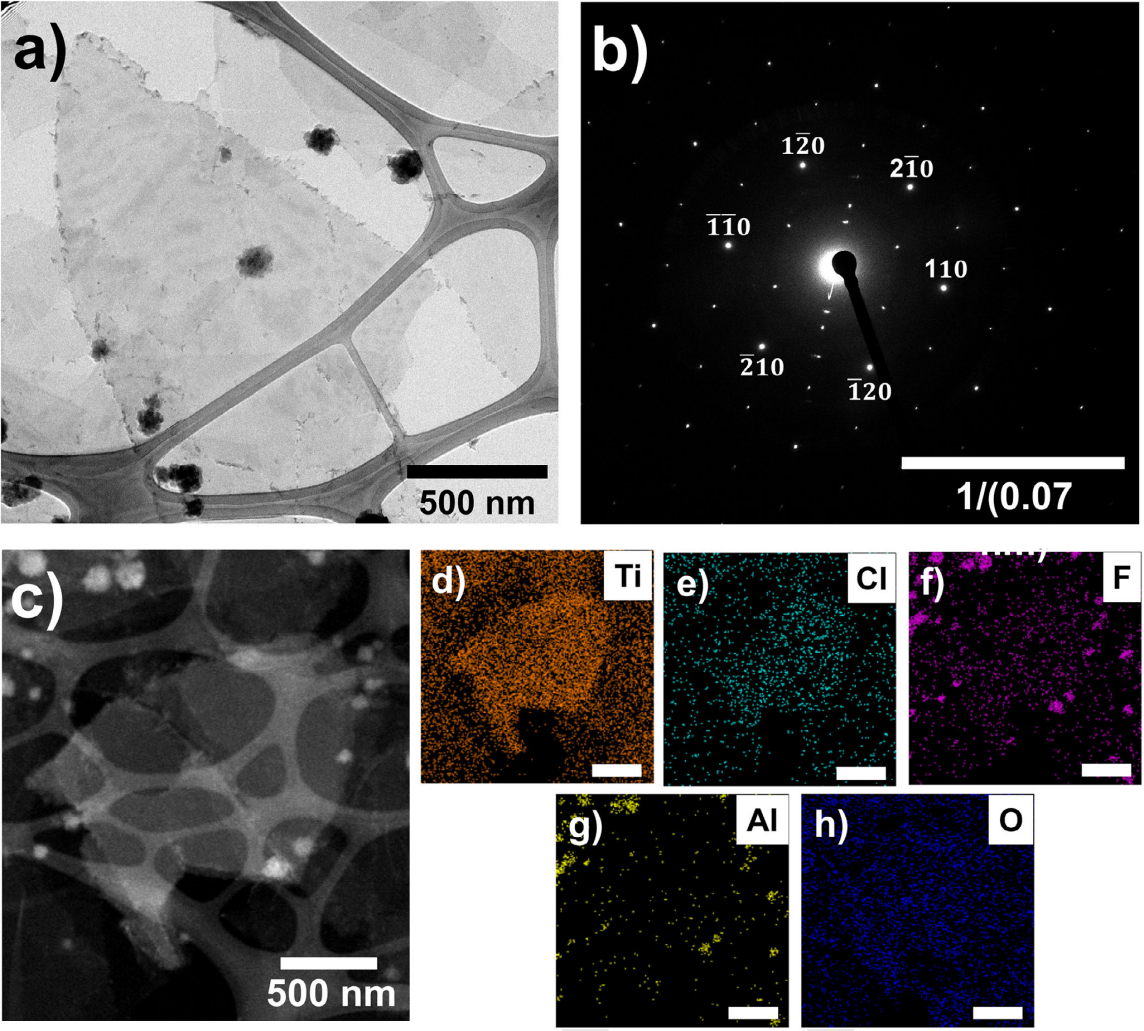
TEM characterization of Ti_3_C_2_T*_x_*: (a) TEM micrograph and corresponding (b) SAED pattern. (c) HAADF‐STEM image with elemental mappings of (d) Ti, (e) Cl, (f) F, (g) Al and (h) O elements. Scale bars in (d–h) represent 500 nm.

To further verify the MXene structure, transmission electron microscopy (TEM) was performed. Figure 2a shows a low‐magnification TEM image of large, typical sheet‐like geometry thin Ti_3_C_2_T*_x_* MXene flakes with well‐defined edges, exhibiting excellent electron transparency. The presence of underlying flakes visible through the top layers confirms the 2D nature of the Ti_3_C_2_T*_x_* MXene and high degree of etching and delamination of the MAX phase. The corresponding selected‐area electron diffraction (SAED) pattern in Figure 2b exhibits well‐defined diffraction spots arranged in hexagonal symmetry. The diffraction pattern can be indexed along the [001] orientation and is consistent with the simulated diffraction pattern of the parent Ti_3_AlC_2_ (ICSD ID 153266), as shown in Figure S3. The simulated pattern matches well with the experimental SAED pattern, confirming that the Ti_3_C_2_T*_x_* MXene retains the crystallinity and hexagonal symmetry of the parent Ti_3_AlC_2_ MAX phase [43], after selective etching of the Al layers and delamination. The indexed reflections clearly show the high‐order planes of the hexagonal lattice.

Additionally, elemental mapping using scanning transmission electron microscopy (STEM) and energy‐dispersive X‐ray spectroscopy (EDS) was carried out to investigate the elemental distribution and surface chemistry of the Ti_3_C_2_T*_x_* MXene (Figure 2c–h). The STEM‐EDS maps confirm the homogeneous distribution of Ti (Figure 2d) throughout the flake, along with the presence of Cl (Figure 2e,f), which are expected surface terminations resulting from the etching process employed in this work. The presence of Al cc. 3.0 at.% (Figure 4g; Table S1) is also detected, likely due to the formation of fluoroaluminate species during the synthesis. The presence of oxygen (Figure 2h; Table S1), corresponding to oxygen terminations (–O), is expected because of oxidation and partial hydrolysis during the etching and washing steps. Furthermore, oxygen can also originate from adsorbed species such as H_2_O, CO_2,_ or hydrocarbons.

Then, to evaluate the optical properties of the synthesized MXene, UV‐Vis spectroscopy was carried out. The Ti_3_C_2_T*_x_* exhibits an absorption minimum at ∼550 nm. A greenish color is sometimes observed in MXene dispersions and is generally associated with partial surface oxidation, which may occur during initial washing steps and handling in aqueous media. For a concentration of ∼0.1 mg/mL, the black line in Figure 1d shows absorption at ∼780 nm. This peak is characteristic of MXene Ti_3_C_2_T*_x_* materials, and it is associated with either an interband transition [44, 45, 46] or surface plasmon [6, 46, 47]. The sample also exhibits broad absorption in the UV region (starts ∼210 nm) with a maximum at ∼390 nm (Figure 1d).

At a concentration of ∼0.01 mg/mL, the red curve still exhibits a peak at ∼778 nm, similar to that observed at higher concentrations. However, two peaks appeared in the UV region at ∼321 nm and ∼272 nm instead of the broad absorption observed for the more concentrated dispersion. This change suggests that oxidation occurs mainly at the surface, and since the dilution causes the delamination of individual layers, unoxidized flakes are released into the solution, resulting in these absorption bands. It means that dilution affects the dispersion's optical response and is likely related to changes in the balance between oxidized and non‐oxidized species, flake aggregation state, and the effective optical contribution of dispersed MXene sheets. The idea of surface‐only oxidation and release of delaminated unoxidized flakes is further supported at an even higher dilution (∼0.001 mg/mL). The surface oxidation becomes negligible compared to the concentration of unoxidized flakes, indicating that the UV‐Vis response is sensitive to both the oxidation and dispersion state of Ti_3_C_2_T*_x_* in solution.

**FIGURE 3:**
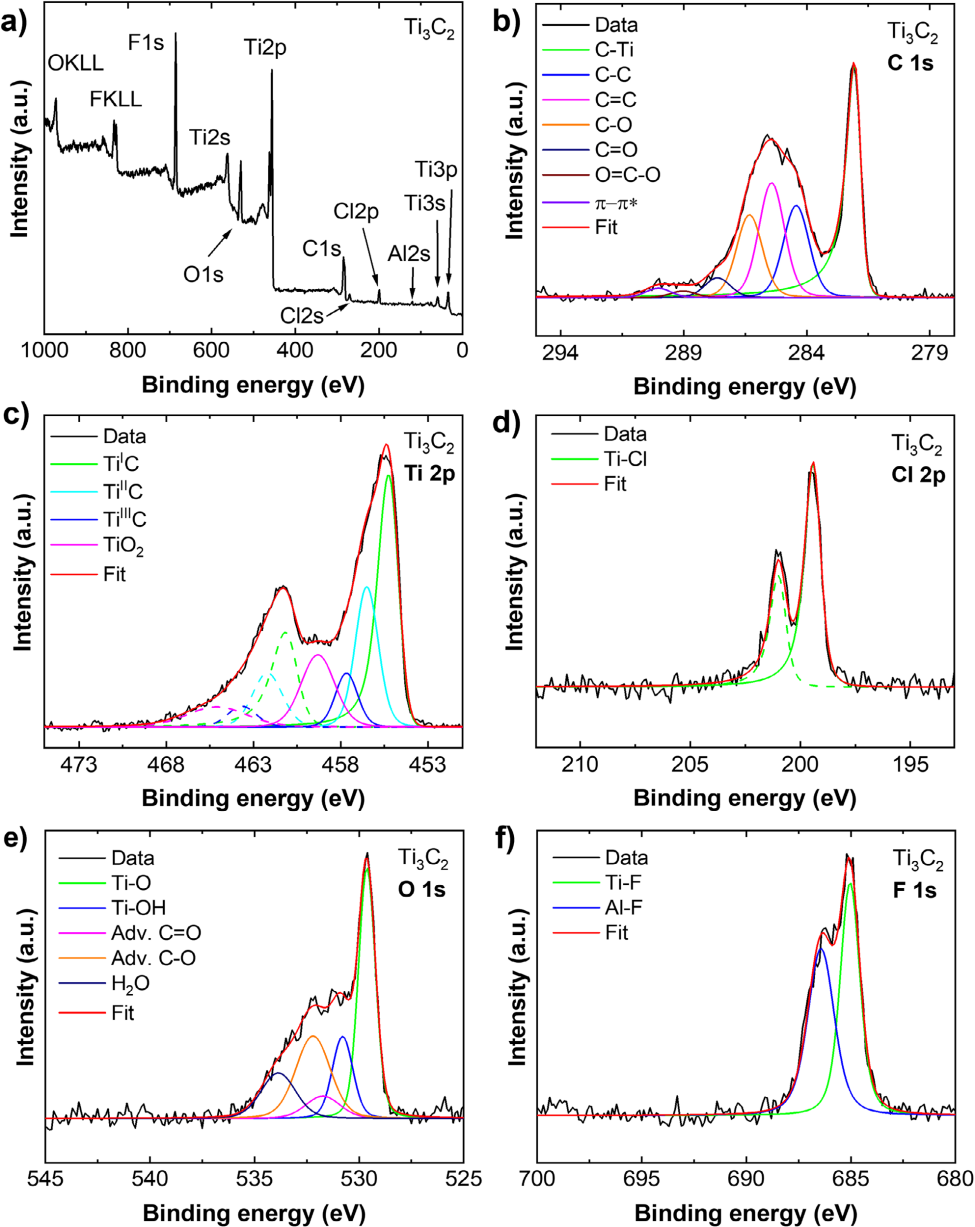
XPS analysis of Ti_3_C_2_T*_x_* MXene: (a) Survey XPS spectrum confirming overall elemental composition. HR spectra of (b) C 1s, (c) Ti 2p, (d) Cl 2p, (e) O 1s, and (f) F 1s core levels.

To complement the structural and optical analysis, X‐ray photoelectron spectroscopy (XPS) was performed on the MXene sample to analyze its surface chemical composition and bonding states. Initially, survey spectra (Figure 3a) were acquired to determine the elemental composition of the Ti_3_C_2_T*_x_* MXene. The sample composition is summarized in Table S2. The data suggest successful exfoliation, as only 2.0 at.% of aluminum (Table S2) was determined on the surface. Since the XRD pattern (Figure 1a) shows no detectable remaining parent MAX phase, and the Al 2p binding energy appears at 76 eV (Figure S4), the residual aluminum can be attributed to adsorbed fluoroaluminate species, such as [AlF_6_]^3−^ [48], on the MXene surface. The elemental composition determined by EDX (Table S1), which shows a residual aluminum amount (3 at.%, Table S1), is consistent with this interpretation.

High‐resolution (HR) XPS spectra were subsequently acquired to provide detailed information on chemical bonding environments. The C 1s (Figure 3b) spectrum was deconvoluted into seven distinct bonding states (Table S3). The primary peak corresponds to the carbidic carbon, indicative of the MXene's core structure, while the remaining components arise from various surface adsorbates such as organic molecules and/or atmospheric CO_2_. The carbidic peak exhibits an asymmetric shape, characteristic of metallic conductors. The Ti 2p spectrum (Figure 3c) was deconvoluted into four bonding states (Table S4) representing increasing oxidation states. The first corresponds to titanium in a pure carbide environment, expected to reside at the center of each MXene layer. The next two states are attributed to surface titanium atoms bonded to terminal groups (Cl, F, O), and the highest‐binding‐energy contribution corresponds to TiO_2_, indicating some degree of surface oxidation.

The HR Cl 2p, O 1s, and F 1s spectra (Figure 3d–f) further support the presence of surface terminations and adsorbed species. The HR O 1s spectrum (Figure 3e) was deconvoluted into five components corresponding to different oxygen environments present on the MXene surface. The peaks at 529.6 eV and 530.8 eV were assigned to Ti–O and Ti–OH surface terminations, respectively. Additional contributions at higher binding energies arise from adsorbed surface species associated with potential surface contamination and interactions with the ambient environment. In particular, the components centered at 533.5 eV and 535.5 eV were attributed to oxygen in C═O and C–O functional groups of adsorbed organic species, while the peak at 533.8 eV corresponds to physisorbed water molecules.

The F 1s high‐resolution spectrum (Figure 3f) was deconvoluted into two components corresponding to fluorine present in different chemical environments. The lower‐binding‐energy (685 eV) contribution is associated with Ti–F surface terminations formed during the etching process. The second component (686.4 eV) is attributed to fluorine bond in fluoroaluminate complex [AlF_6_]^3−^, which may remain after etching and can be adsorbed on the MXene surface or trapped between its layers. The relative contents of the identified Ti bonding states and surface terminations/species are summarized in Tables S4 and S5, respectively. Together, these results confirm a chemically terminated Ti_3_C_2_T*_x_* surface containing O‐, OH‐, F‐, and Cl‐associated species.

**FIGURE 4:**
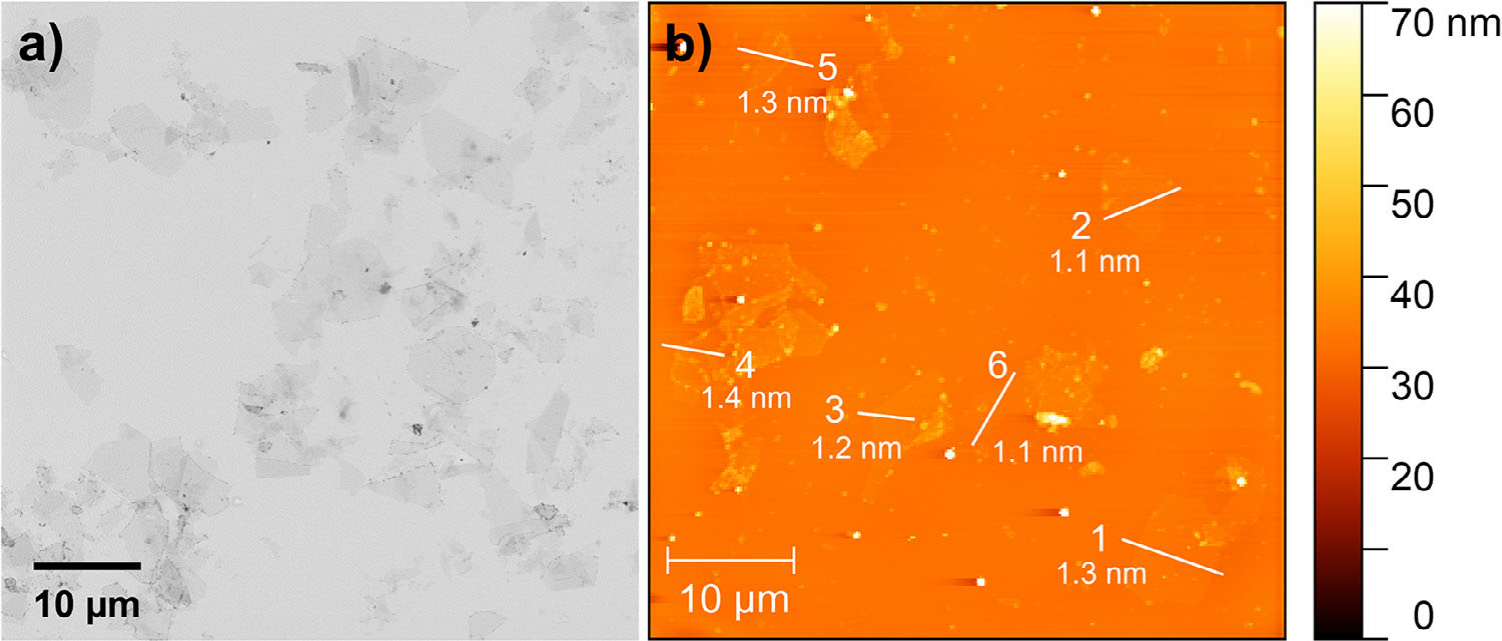
STEM and AFM characterization of Ti_3_C_2_T*_x_* MXene: (a) STEM image showing exfoliated MXene flakes; (b) AFM height image with representative MXene flakes and corresponding thickness values, consistent with single‐layer MXene. The corresponding AFM line profiles used to determine the thickness are provided in Figure S4.

To further examine the morphology and thickness of individual MXene nanosheets, scanning transmission electron microscopy (STEM) and AFM analysis were performed (Figure 4). The STEM image (Figure 4a) reveals thin, transparent Ti_3_C_2_T*_x_* MXene flakes and uniform contrast across the nanosheets. More than 20 flakes were examined, all exhibiting consistent single‐layer contrast with no evidence of multilayer stacking, confirming the successful delamination of the layered structure. The thickness of the MXene flakes was evaluated by AFM. The AFM height image (Figure 4b) shows representative MXene flakes. The average thickness of the selected 6‐line profiles is in the range of 1‐1.4 nm, consistent with that of single‐layer Ti_3_C_2_T*_x_* MXene. The corresponding AFM line profiles used to determine the thickness are provided in Figure S5. The consistent thickness observed via AFM directly validates that this hydrothermal‐assisted etching process with in situ intercalation effectively achieves direct delamination to single layers [24, 49].

The electrical conductivity of the prepared MXene film was calculated to be 7500 S cm^−^^1^ from the measured film thickness (430 nm), determined by the AFM scratch method (Figure S6a), and the sheet resistance (3.1153 Ω sq^−^^1^) measured by the four‐point probe method (Figure S6b). This result indicates that the synthesized MXene exhibits excellent electrical conductivity and a compact structure, enabling efficient charge transfer across the boundaries between individual flakes. This can be attributed to the uniform etching of the MAX phase and the formation of a more ordered structure with fewer defects [9, 50]. The electrical conductivity obtained in this study is consistent with the higher range of conductivities typically reported for MXene films and is compared with the literature in Table 1. For example, MXene films synthesized by electrodeposition exhibit 7400 S cm^−^^1^ [51]; similarly, freestanding films produced using the MILD method typically show moderate conductivities of 900–4400 S cm^−^^1^ [9, 13], also, films synthesized using high‐pressure homogenization and HF etching followed by calcination result in poor flake alignment and residual inclusions, showing much lower conductivities of about 1500 S cm^−^^1^ and 2140 S cm^−^^1^ [52, 53], respectively. Importantly, the present work provides not only a shortened hydrothermal synthesis route (6 h) with in situ Li^+^ intercalation, but also quantitative performance metrics, including MXene yield (74.2% ± 3.7%) and electrical conductivity (7500 S cm^−^^1^). For previously reported MILD synthesis routes, including LiCl‐assisted and hydrothermal approaches, yield and/or electrical conductivity data are not always available, thereby limiting a direct assessment of their overall effectiveness relative to the present method.

**TABLE 1:** Comparison of synthesis methods, intercalants, reaction conditions, yield, and electrical conductivity of Ti_3_C_2_T*_x_* MXene reported in the literature with the Ti_3_C_2_T*_x_* MXene synthesized in this work.

Method	Intercalant	Conditions	Yield (%)	Electrical conductivity (S/cm)	Refs.
Hydrothermal‐assisted in situ intercalation	LiCl	6 h, 90°C	74	7 500	This work
2 g LiF, 20 mL 12.2 M HCl (MILD)	—	24 h, 40°C	—	900	[9]
HF etching + in situ intercalation	LiCl	24 h, 25°C	—	—	[39]
High‐pressure homogenization	—	36–48 h	80	1 500	[52]
0.66 g LiF, 10 mL 6 M HCl (MILD)	—	45 h, 40°C	—	1 500	[13]
HF, Calcination at 600°C	Alkali treatment with NaOH solution	40 h, RT	—	2 140	[53]
1.6 g LiF, 20 mL 9 M HCl (MILD)	LiCl	40 h, RT	20	4 400	[54]
Electrode deposition	—	72 h stirring	—	7 400	[51]
2 g LiF, 9 M HCl, (MILD)	Cyclic ultrasonic‐centrifugal separation	48 h, 35°C	72	—	[31]
Hydrothermal‐assisted intercalation	TMAOH	24 h, 140°C	74	—	[24]

## 3 Conclusion

Ti_3_C_2_T*_x_* MXene has been etched and delaminated from the corresponding MAX phase using a facile, time‐efficient synthesis method, compared to direct and in situ HF etching. In this proposed method, the Ti_3_AlC_2_ MAX phase was etched with HCl/LiF mixture in a hydrothermal autoclave at 90°C for 6 h. This process takes eight times less than conventional etching methods, which can take up to 48 h. Moreover, this approach enables direct delamination of MXenes via in situ intercalation, further reducing post‐etching processing time. The method's efficacy was confirmed using various characterization techniques. XRD pattern confirms the formation of Ti_3_C_2_T*_x_* MXene and indicates almost complete etching of Al and a high degree of delamination of MXene sheets. XPS surface‐composition results complemented the XRD findings, showing the presence of typical elements such as oxygen, carbon, and titanium, along with fluorine and chlorine, and notably distinguishing the etched product from the precursor. Moreover, TEM with corresponding elemental mapping also confirmed the formation of high‐quality MXene with exfoliated, layered structures. This research holds importance that extends well beyond its initial findings. This method offers the potential to create a wide array of 2D MXene structures from over 300 known MAX phases and could enable large‐scale, time‐efficient production of high‐quality MXenes.

## 4 Experimental Section

### 4.1 Materials

Titanium aluminum carbide (Ti_3_AlC_2_, Mesh 400, ≤40 µm particle size, > 90% pure) was purchased from Sigma‐Aldrich. The etching solvents were lithium fluoride (LiF, >99 wt.%, Merck, Darmstadt, Germany), hydrochloric acid (HCl, 37 wt.%, Merck, Darmstadt, Germany), and the intercalant was lithium chloride (LiCl, 99 wt.%, Merck, Darmstadt, Germany). Deionized (DI) water was used for washing.

### 4.2 Synthesis

**SCHEME 1:**
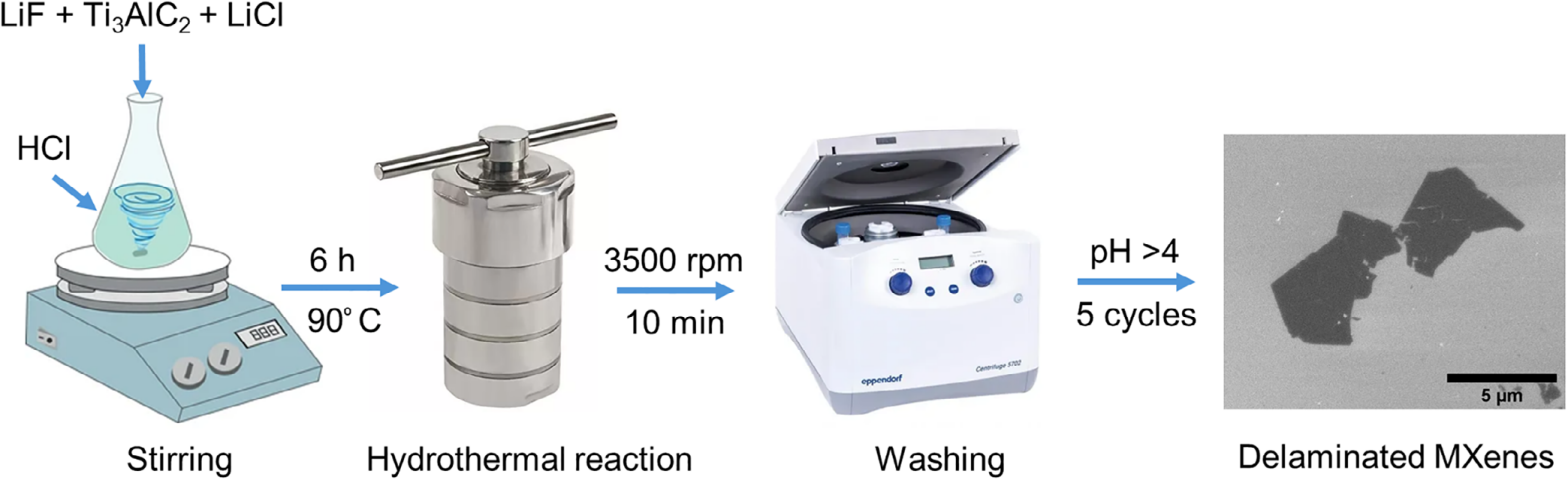
Schematic illustration of Ti_3_C_2_T*_x_* MXene synthesis from MAX phase precursor.

Ti_3_C_2_T*_x_* was synthesized as shown in the schematic diagram (Scheme 1). Firstly, 3.2 g of LiF was slowly added to 40 mL of 37% HCl, and the mixture was stirred for 30 min at room temperature. Further, 2 g of Ti_3_AlC_2_ MAX phase was added slowly to the mixture under stirring for 30 min. Then, 2 g of LiCl was added to the solution, and the mixture was stirred again for 30 min. Afterwards, the mixture was transferred to a Teflon‐lined hydrothermal autoclave and heated at 90°C for 6 h, then cooled to room temperature. The mixture was centrifuged at 3500 rpm to remove the acids. The liquid was discarded, and 200 mL of fresh DI water was added to the solid, which was shaken for 15 min and centrifuged. The process was repeated until a pH> 4 was achieved. Then, 200 mL of fresh DI water was added, shaken for 10 min, centrifuged, and the liquid was collected until a total of 3.6 L of suspension was obtained. The collection was stopped at this point as no further dark solution was being extracted. The remaining solid was also collected and dried.

### 4.3 Etching Mechanism

The hydrothermal etching process occurs in an aqueous LiF/LiCl medium inside a Teflon‐lined autoclave. High temperatures in hydrothermal reactions typically facilitate the dissolution of materials that are otherwise difficult to dissolve under normal conditions [22]. In this system, LiF reacts with HCl to generate fluoride‐containing etching species according to Equation (1), which then selectively react with the Al layers in Ti_3_AlC_2_, as represented in Equation (2):

(1) LiF+HCl→LiCl+HF

(2) $$Ti_{3}\mathrm{Al}C_{2}+3\mathrm{HF}\;\to Ti_{3}C_{2}+\mathrm{Al}F_{3}+3/2H_{2}$$

The HF reacts with Al to form AlF_3_ and gaseous H_2_, while the Ti_3_C_2_T*_x_* 2D layered structure is released from the parent MAX phase. The enclosed hydrothermal environment facilitates ionic mobility, diffusion, and overall reaction kinetics, thereby accelerating the transformation of Ti_3_AlC_2_ into Ti_3_C_2_T*_x_*. At the same time, the closed chamber helps minimize the loss of volatile etching species and supports a more homogenous reaction environment than open‐system processing. The hydrothermal conditions also promote the dissolution of AlF_3_ and help prevent secondary reactions that might occur in open systems [9]. In addition, the enclosed reaction environment may reduce undesirable oxidation of titanium to TiO_2_ during the reaction.

**SCHEME 2:**
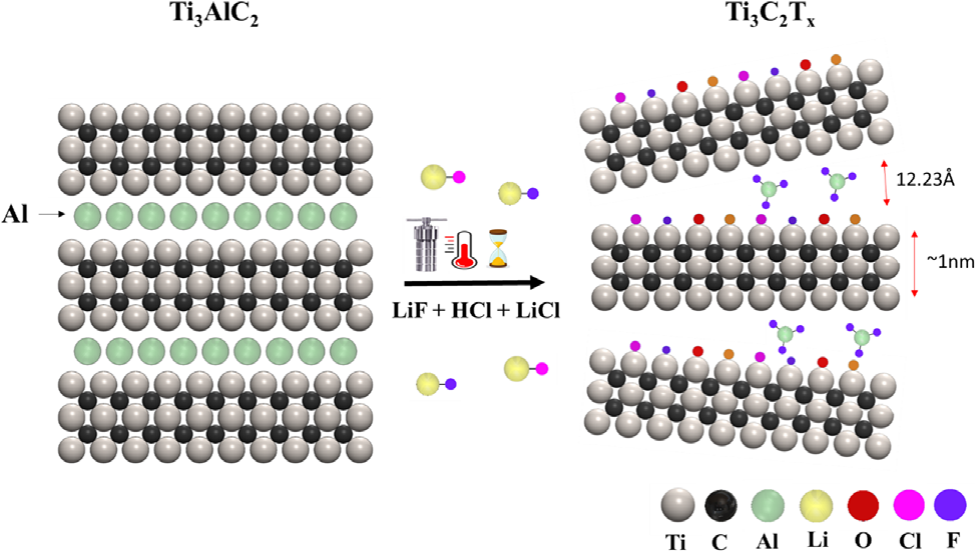
Schematic illustration of the proposed etching and in situ intercalation‐assisted delamination mechanism of Ti_3_AlC_2_ MAX into single‐layered Ti_3_C_2_T*_x_* MXene under hydrothermal LiF/HCl conditions.

The addition of LiCl in the reaction serves as an in situ intercalant, providing additional Li^+^ ions that aid exfoliation and increase interlayer spacing, thereby improving the yield of few‐layer 2D MXene sheets [55]. Without the need for a separate post‐etching intercalation step, this assists layer separation and enhanced intercalation by weakening the van der Waals forces between MXene sheets, thus facilitating the exfoliation of MXenes into single‐layer 2D MXenes [35]. Additionally, the presence of Li^+^ also leads to a greater structural order in the MXene [39]. Scheme 2 presents Li^+^ ion intercalation into MAX layers.

### 4.4 Materials Characterization

#### 4.4.1 X‐Ray Diffraction (XRD)

XRD pattern of MXene was acquired using a Bruker D8 Advance instrument (Bruker AXS GmbH). The instrument was configured in Bragg–Brentano parafocusing geometry and employed a CuK*α* radiation source (λ = 0.15418 nm), operating at *U* = 30 kV and *I* = 10 mA. The XRD pattern was collected at room temperature over 2θ values from 5 to 90° with a step size of 0.02°. Data analysis was conducted using the HighScore Plus 4.9 software package. For sample preparation, the Ti_3_C_2_T*_x_* MXene suspension was drop‐cast onto a Si/SiO_2_ substrate and then dried at room temperature. The Ti_3_AlC_2_ MAX phase was measured directly in powder form as received on a Si/SiO_2_ substrate.

#### 4.4.2 Raman Spectroscopy

An InVia Raman microscope (Renishaw, England) was used for Raman spectroscopy measurements in backscattering geometry with a CCD detector. A Cobolt 08 narrow‐linewidth diode laser (785 nm, 120 mW) with an 830‐line mm^−1^ diffraction grating, an applied power of 1.25%, and a 20× objective was used for the measurements. At room temperature, the Raman spectrum was collected with 10 accumulations and 10 s of exposure time in the range of 100–1800 cm^−1^. For instrument calibration, a peak at 520 cm^−1^ was obtained on a silicon reference before the measurement. The XRD sample was used for the Raman measurement.

#### 4.4.3 Scanning Electron Microscopy (SEM), Transmission Electron Microscopy (TEM) and Scanning Transmission Electron Microscopy (STEM)

The morphology of MXene flakes was characterized by SEM, TEM and STEM. SEM images were obtained using an S‐3400N (Hitachi, Tokyo, Japan) at an accelerating voltage of 5 kV. Silicon wafers (1 × 1 cm) were used as substrates for nanocoating. For sample preparation, the suspension was drop‐cast on a silicon substrate and dried at 60°C in an oven. SEM images of the leftover paste were obtained with the same instrument at an accelerating voltage of 3 kV. The paste was drop‐cast onto a glass substrate and dried at 60°C in an oven. TEM was used to analyze the morphology and structure of Ti_3_C_2_T*_x_* MXenes using a JEOL 2200 FS microscope (Jeol, Akishima, Japan) equipped with a TVIPS camera, operating at 200 kV. 3 µL of the prepared suspension was drop‐cast onto a TEM grid (Cu, 300 mesh, Lacey Carbon from TED PELLA, Inc.) and dried in air. The simulated electron diffraction pattern used to index the experimental SAED pattern was generated from the Ti_3_AlC_2_ crystal structure (ICSD ID 153266) using CrystalMaker and SingleCrystal software. STEM imaging was conducted using a TESCAN MAIA 3 system (Tescan Brno, s.r.o., Czech Republic) operated in STEM mode at 30 kV. 3 µL of the prepared suspension was drop‐cast onto a TEM grid (Cu, 300 mesh, Lacey Carbon from TED PELLA, Inc.) and dried in air.

#### 4.4.4 X‐Ray Photoelectron Spectroscopy (XPS)

High‐resolution (HR)‐XPS was performed using a SPECS spectrometer (Specs, Germany) equipped with a monochromatic Al Kα X‐ray radiation source (1486.7 eV) and a hemispherical electron analyzer Phoibos 150. The samples were placed on a conductive carbon tape and compensated with a flood gun to yield a C 1*s* peak at 284.8 eV. The survey spectra were recorded with E_p_ set to 100 eV, and the core lines' high‐resolution (HR) spectra with E_p_ set to 20 eV. HR scans were performed for C 1s, O 1s, Ti 2p, Al 2p, F 1s, and Cl 2p core levels. The base chamber pressure during the acquisitions was 10^−9^ mbar or lower. Due to heavy charge development on the samples' surface, a low‐energy electron flow generated by an electron flood gun has been used to compensate for the charge buildup. The CasaXPS software package was used to quantify and curve‐fit core‐level spectra, using Shirley‐type backgrounds.

#### 4.4.5 Atomic Force Microscopy (AFM)

AFM measurements were carried out on a Ntegra Spectra from NT‐MDT. The surface scans were performed in tapping (semi‐contact) mode, and a cantilever with a strain constant of 1.5 kN·m−1, equipped with a standard silicon tip with a curvature radius of less than 10 nm, was used for all measurements. For sample preparation, the Ti_3_C_2_T_x_ MXene suspension was deposited onto freshly cleaved mica substrates via spin coating and subsequently dried under ambient conditions. The measurements were performed under ambient conditions with a scan rate of 1 Hz and 512 scan lines. Moreover, AFM (NanoWizard3, NanoScience, JPK Instruments AG (Germany) was used to measure the thickness of the MXene coating prepared for electrical conductivity measurements. For these measurements, the coating was prepared by drop‐casting the MXene suspension onto a glass Petri dish, which was then dried at 60°C.

#### 4.4.6 Ultra‐Violet Visible (UV‐Vis) Spectroscopy

A PerkinElmer Lambda 850+ spectrometer coupled with an integrating sphere was used for the UV‐Vis measurements. Tungsten/deuterium lamp (200–850 nm) and 0.2 s nm^−1^ integration time were used. A quartz glass cuvette with a length of 1 cm was filled with MXene suspension prepared before the measurement by dilution with distilled water of the stock suspension of MXene (0.557 mg/mL), resulting in concentrations ∼0.1 mg/mL, ∼0.01 mg/mL, and ∼0.001 mg/mL.

#### 4.4.7 Four‐Probe Method

An Ossilia four‐point probe (±100 µV—±10 V) was used to measure the sheet resistance of the prepared sample by drying on a glass surface at 60°C. A minimum of 3 points was used to measure sheet resistance in Ω/Sq.

#### 4.4.8 Electrical Conductivity Calculation

Electrical conductivity was calculated with the expression Conductivity (σ) = 1/(*R_s_* × Thickness), where σ is the electrical conductivity (S/cm), *R_s_* is the sheet resistance (Ω/square), and the thickness (*t*) of the sheet is in cm [56].

## Author Contributions

**Muhammad Imran Rameel**: methodology; validation; formal analysis; investigation; data curation; writing – original draft; visualization. **Gediminas Monastyreckis**: conceptualization; methodology; validation; investigation; data curation; writing – review & editing; visualization. **Filipa M. Oliveira**: methodology; validation; investigation; formal analysis; data curation; writing – review & editing; visualization; supervision; project administration; funding acquisition. **Bing Wu**: methodology; investigation; formal analysis; data curation; writing – review & editing; visualization. **Jiří Šturala**: methodology; investigation; formal analysis; data curation; writing – review & editing; visualization. **Vlastimil Mazánek**: methodology; investigation; formal analysis; data curation; writing – review & editing; visualization. **Zdeněk Sofer**: validation; writing – review & editing; supervision; project administration. **Daiva Zeleniakiene**: conceptualization; methodology; validation; investigation; resources; writing – review & editing; supervision; project administration; funding acquisition.

## Funding

Grant Agreement No. 101182521. Project No. 25‐16769S from the Czech Science Foundation. Project LL2101 from the Ministry of Education Youth and Sports (MEYS). Project Advanced Functional Nanorobots (reg. No. CZ.02.1.01/0.0/0.0/15_003/0000444) financed by the EFRR. Project No. CZ.02.01.01/00/22_008/0004558 supported by the MEYS.

## Conflicts of Interest

The authors declare no conflicts of interest.

## Supporting information




**Supporting File**: smtd70871‐sup‐0001‐SuppMat.pdf.

## Data Availability

The datasets generated and/or analyzed during the current study are accessible via the Zenodo repository: https://zenodo.org/records/17787048.
