# Supplementary material for: Hydrothermal‐Assisted In Situ Intercalation Synthesis of Ti3C2Tx MXene in 6 Hours
Source: Small Methods. 2026 Jul 28;10(16):e70871. doi: 10.1002/smtd.70871 (PMC13505694; doi:10.1002/smtd.70871)
Supplement: Supplementary file 1 [file SMTD-10-e70871-s001.pdf]

Supporting Information

Hydrothermal-Assisted *in situ* Intercalation Synthesis of $Ti_3C_2T_x$ MXene in 6 hours

Muhammad Imran Rameel, Gediminas Monastyreckis, Filipa M. Oliveira,* Bing Wu, Jiří Šturala, Vlastimil Mazánek, Zdeněk Sofer,* and Daiva Zeleniakiene*

Muhammad Imran Rameel, Gediminas Monastyreckis, and Daiva Zeleniakiene
Department of Mechanical Engineering, Kaunas University of Technology, Studentu 56, 51424 Kaunas, Lithuania
E-mail: muhammad.rameel@ktu.edu

Filipa M. Oliveira, Bing Wu, Jiří Šturala, Vlastimil Mazánek, and Zdeněk Sofer
Department of Inorganic Chemistry, Faculty of Chemical Technology, University of Chemistry and Technology Prague, Technická 5, 166 28 Prague 6, Czech Republic
E-mail: filipa.oliveira@vscht.cz, zdenek.sofer@vscht.cz

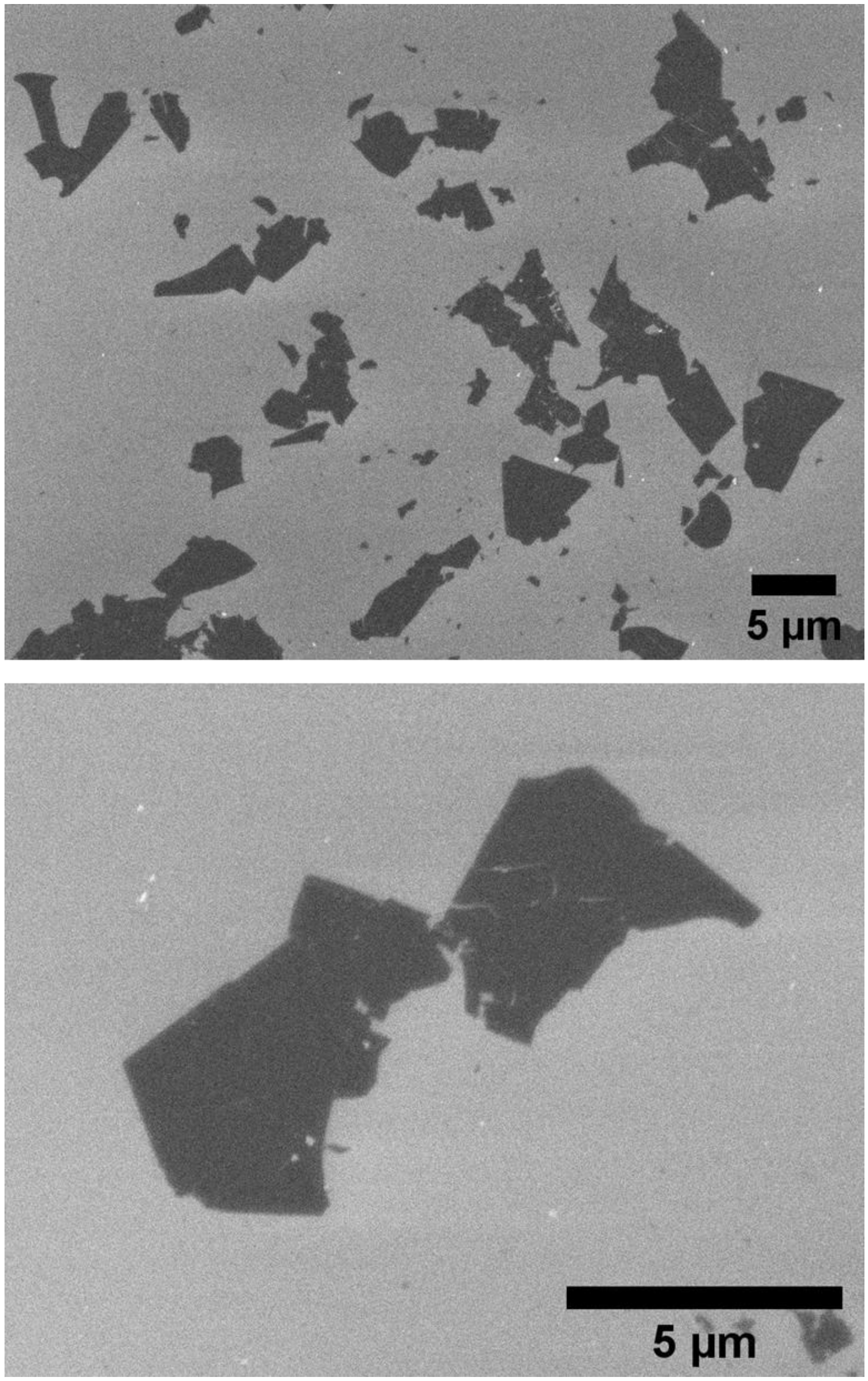

Figure S1: Additional SEM micrographs of $Ti_3C_2T_x$ MXene flakes synthesized via the hydrothermally assisted *in situ* intercalation strategy, 90 °C for 6 h.

2b).

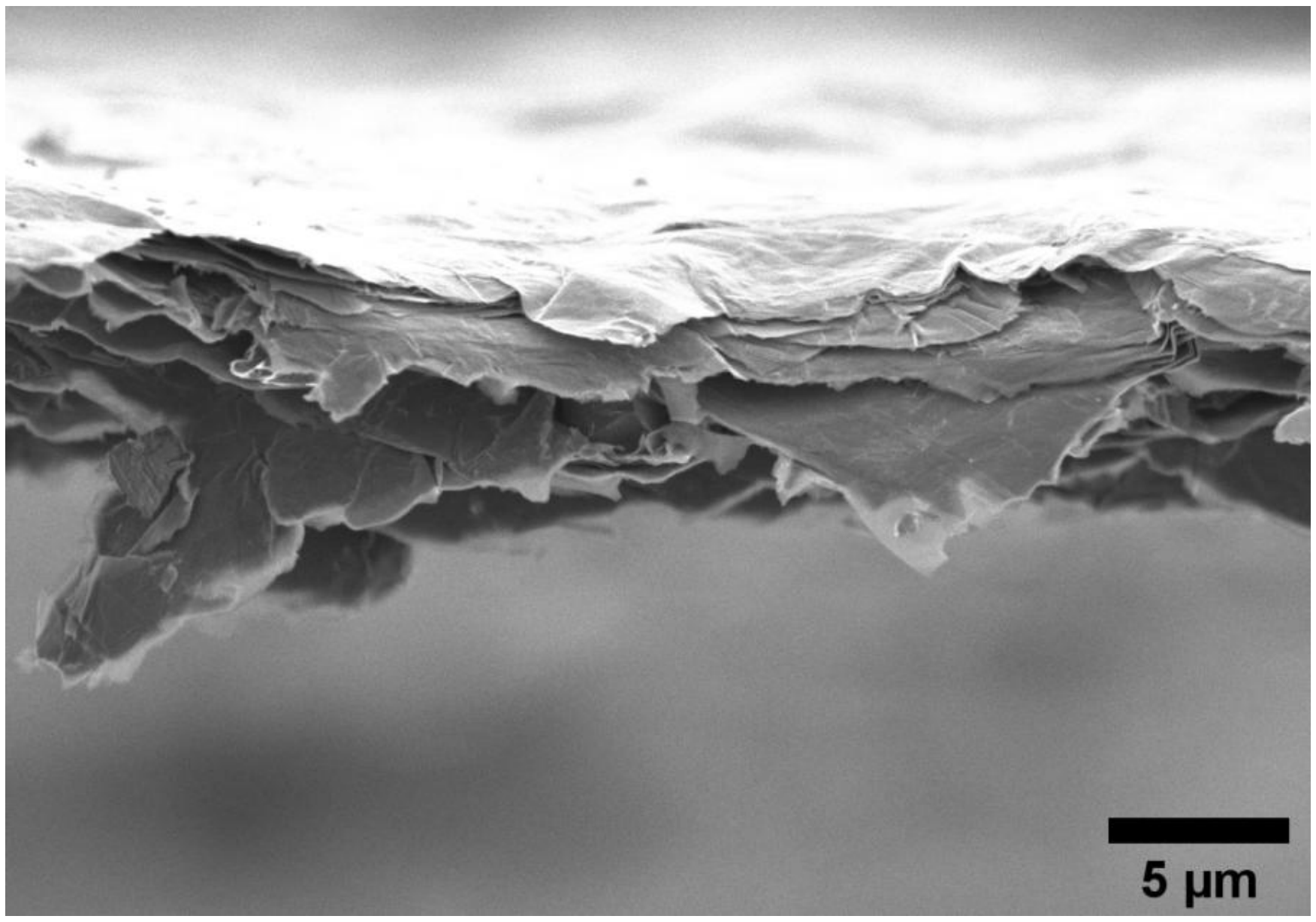

Figure S2: SEM micrograph of leftover $Ti_3C_2T_x$ MXene paste.

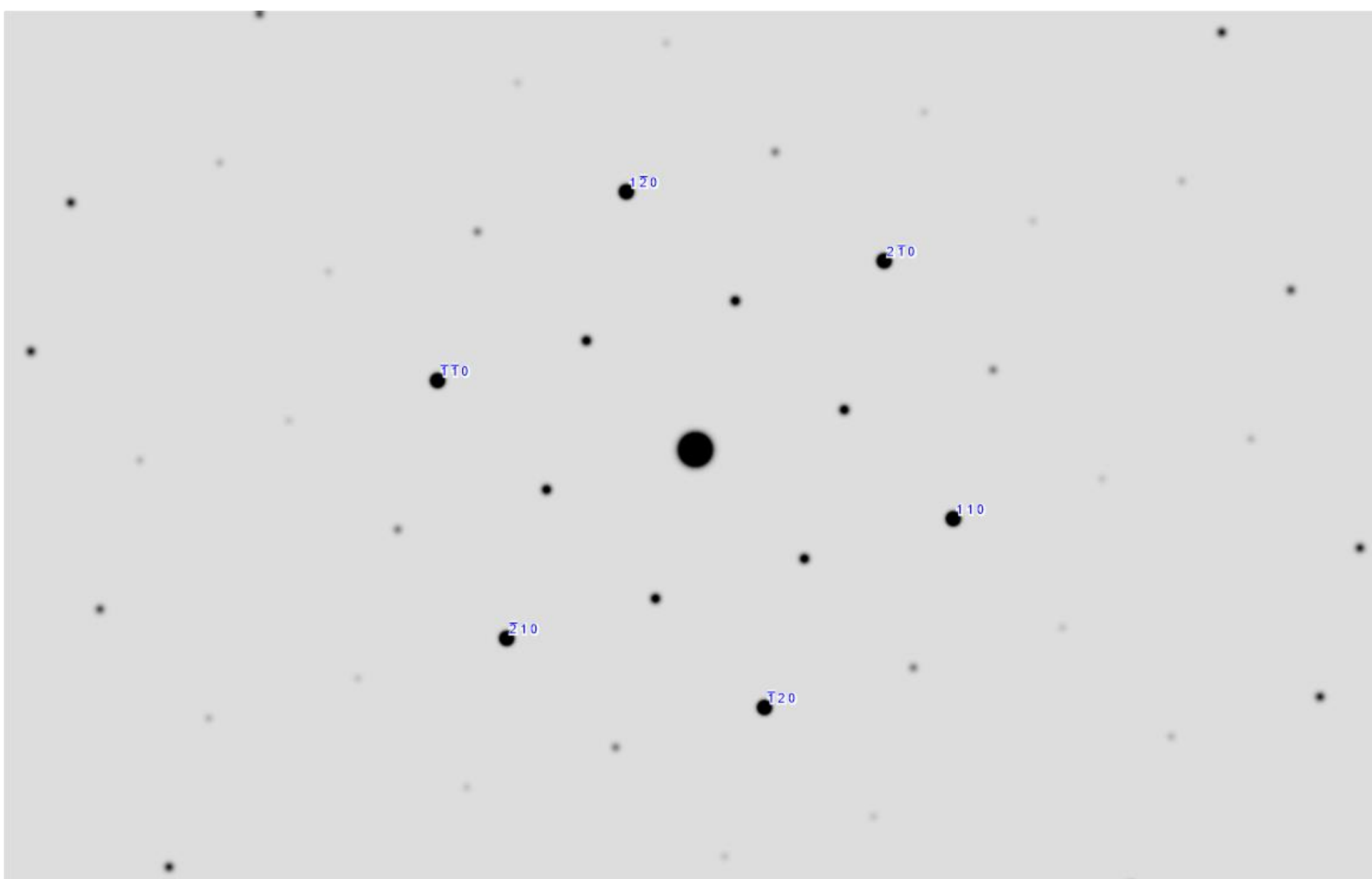

Figure S3: Simulated electron diffraction pattern of Ti_3AlC_2 crystal structure along [001] axis generated from crystallographic data (ICSD ID 153266). The indexed reflections correspond to the hexagonal lattice planes observed in the experimental SAED pattern of $Ti_3C_2T_x$ MXene (Figure 2b).

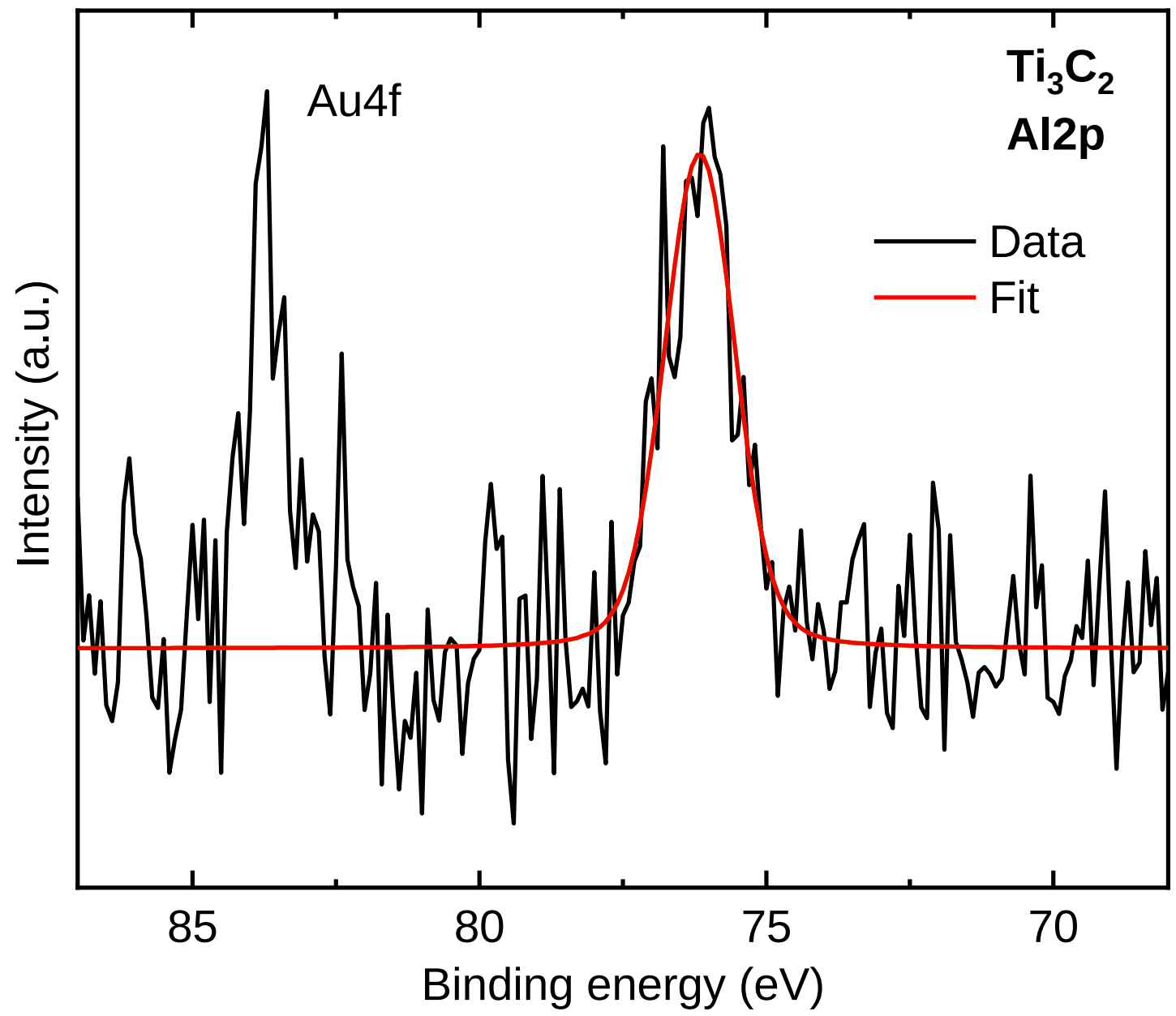

Figure S4: XPS analysis of $Ti_3C_2T_x$ MXene: HR spectra of Al 2p core level.

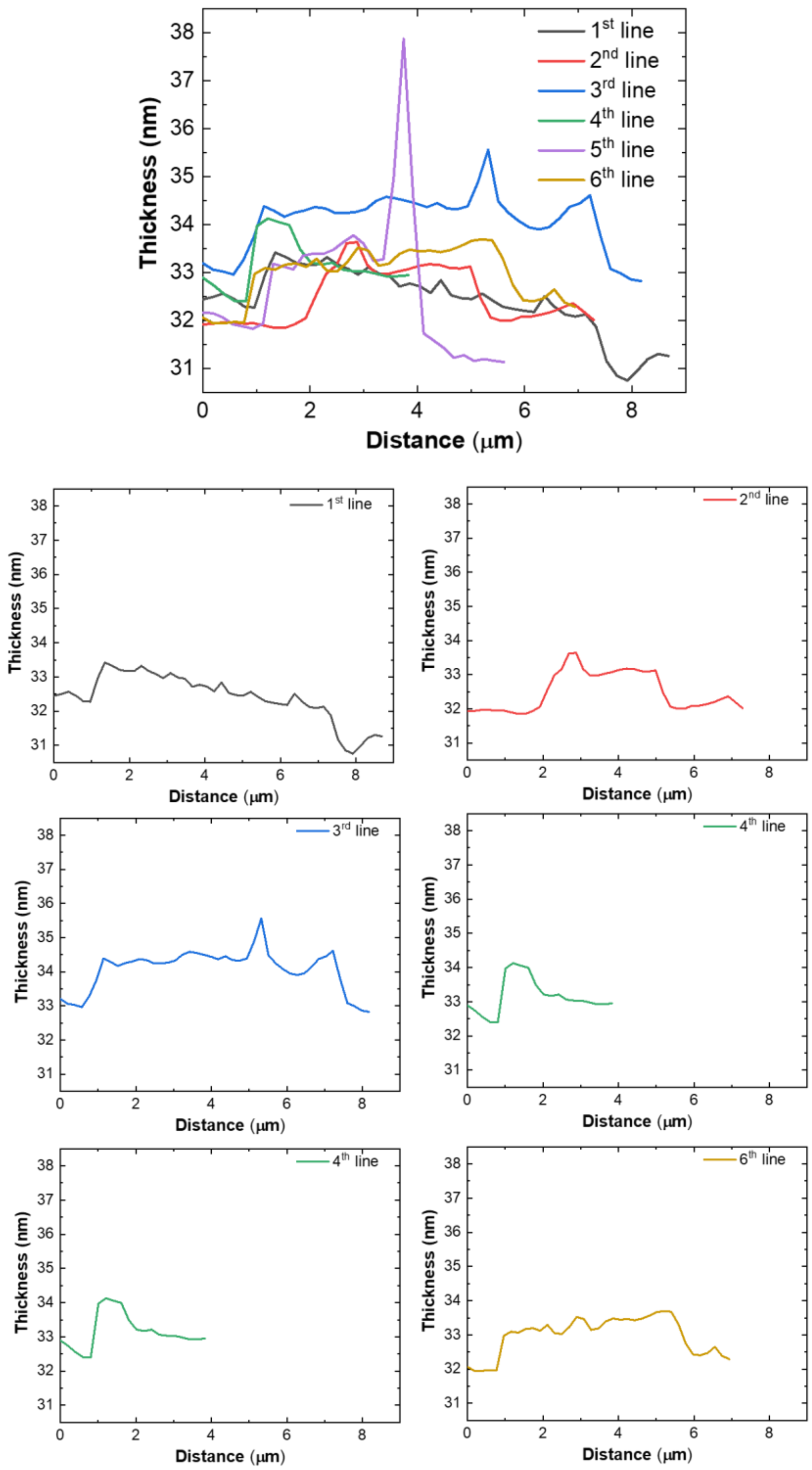

Figure S5: AFM height profiles corresponding to the $Ti_3C_2T_x$ MXene flakes marked in Figure 4b. The line profiles were used to extract the heights of the MXene flakes, yielding thickness values of ~1.1-1.4 nm.

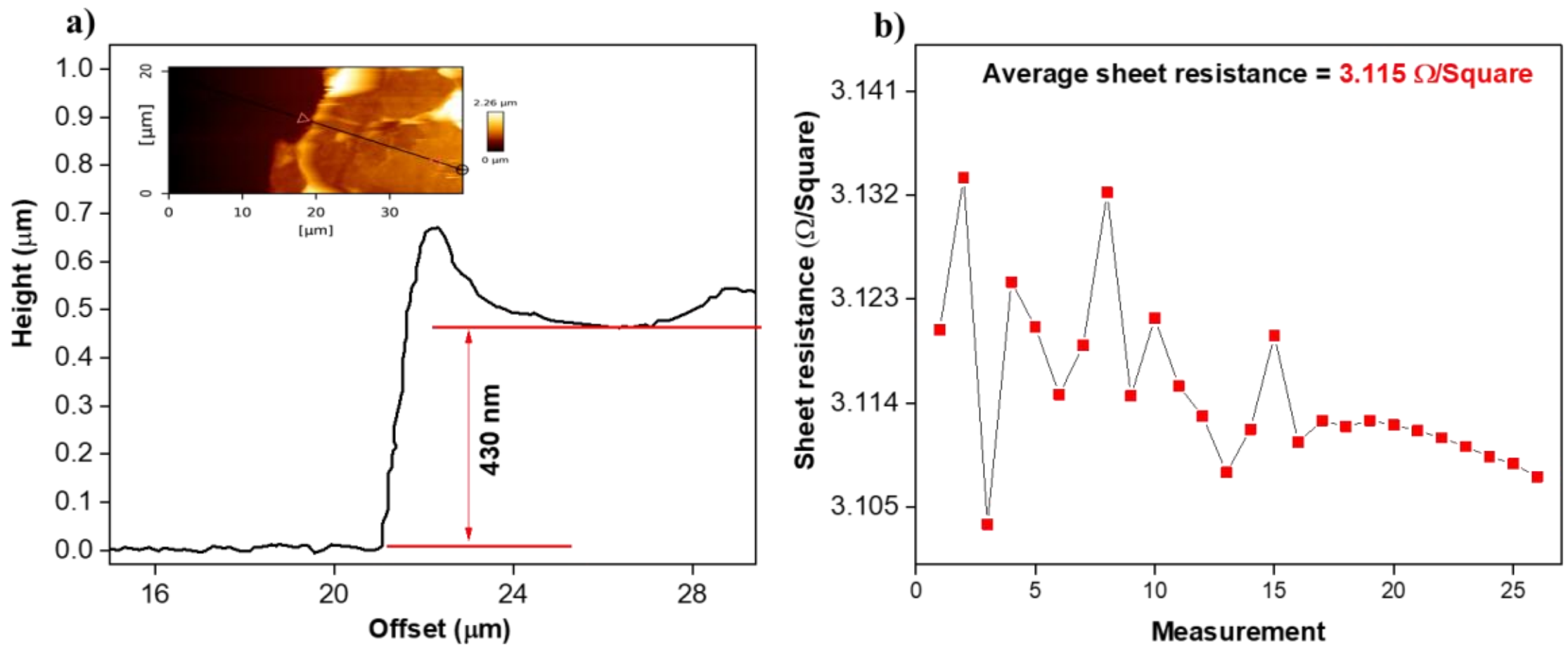

Figure S6: Characterization of $Ti_3C_2T_x$ MXene: a) AFM line scan and corresponding height profile showing the cross-sectional thickness of a $Ti_3C_2T_x$ MXene sheet. b) Sheet resistance of the MXene sheet measured with the four-point probe method.

Table S1: Elemental composition (in at. %) of $Ti_3C_2T_x$ MXene by EDS analysis from TEM measurements.

**Element**	F	O	Ti	Cl	Al
**Composition**	21.9	36.8	33.3	5.0	3.0

Table S2: Surface chemical composition of $Ti_3C_2T_x$ MXene obtained by XPS in at.%.

**Element**	F	O	Ti	C	Cl	Al
**Composition**	18.9	17.8	18.8	38.0	3.3	2.0

Table S3: Individual concentration (in at. %) of bonding states in $Ti_3C_2T_x$ MXene obtained by fitting of HR C 1s XPS spectrum.

**Bonding state**	Carbide	C-C	C=C	C-O	C=O	O=C-O	π-π*
**Binding energy (eV)**	282.1	284.5	285.5	286.3	287.6	289.0	290
**Concentration**	36.8	17.9	22.3	16.1	3.9	1.3	1.7

Table S4: Individual concentration (at. %) of bonding states in $Ti_3C_2T_x$ MXene obtained by fitting of HR Ti 2p XPS spectrum.

**Bonding state**	$Ti^{I}C$	$Ti^{II}C$	$Ti^{III}C$	TiO_2
**Binding energy (eV)**	455.3	456.5	457.7	459.3
**Concentration**	44.8	26.9	8.9	19.4

Table S5: Individual concentration (at. %) of identified surface terminations in $Ti_3C_2T_x$ MXene, as estimated from the fitted components of the deconvoluted HR Cl 2p, O 1s and F 1s XPS spectra.

**Terminal group**	Cl	O	OH	F
**Concentration**	16	11	27	46
